# Prolonged incubation time unwarranted for acute periprosthetic joint infections

**DOI:** 10.1128/jcm.01143-24

**Published:** 2025-01-16

**Authors:** E. R. L. Morreel, H. A. van Dessel, J. Geurts, P. H. M. Savelkoul, I. H. M. van Loo

**Affiliations:** 1 Department of Medical Microbiology, Infectious Diseases and Infection Prevention, Maastricht University Medical Centre 199236 , Maastricht, the Netherlands; 2 Care and Public Health Research Institute (CAPHRI), Maastricht University 168092 , Maastricht, the Netherlands; 3 Department of Orthopedic Surgery, Maastricht University Medical Centre (MUMC+) 199236 , Maastricht, the Netherlands; Johns Hopkins University 1466 , Baltimore, Maryland, USA

**Keywords:** periprosthetic joint infection, PJI, time to diagnosis, acute, chronic, enriched media, blood culture bottle, broth, sonication, culture

## Abstract

Current laboratory protocols for periprosthetic joint infections (PJIs) involve a standard 10- to 14-day incubation period. However, recent evidence indicates considerable variability in the time to diagnosis (TTD) between acute and chronic PJIs. TTD is also influenced by the employed culture media and sample types. Enriched liquid media, such as broths and blood culture bottles, along with sonication fluid culture, are commonly used, though their incremental benefit for PJI diagnosis remains debated. We retrospectively analyzed 187 confirmed hip and knee PJIs, each with at least three intraoperative samples. Comparison of TTD among early acute (*n* = 68), late acute (*n* = 52), and late chronic (*n* = 67) PJIs revealed a significant difference, particularly between late acute and late chronic infections (*P* < 0.004). Early acute and late acute PJIs were diagnosed within 5 days in 97.1% and 98.1% of cases, respectively, contrasting with 14 days required for 97.1% of late chronic PJIs. Enriched liquid media significantly improved species detection, especially in polymicrobial and anaerobic infections. Pediatric and anaerobic blood culture bottles demonstrated superior efficacy over thioglycolate broths for diagnostic confirmation. Sonication fluid culture was essential for confirming diagnoses in 17.6% of cases. Our findings highlight that clinical presentation, rather than time since primary arthroplasty, should guide incubation duration: both early acute and late acute PJIs can be diagnosed within 5 days. Medical microbiology laboratories should consider shorter incubation times for acute PJIs to optimize diagnostic efficiency. The use of blood culture bottles and sonication fluid culture proves invaluable for accurate PJI diagnosis.

**IMPORTANCE:**

While molecular techniques are becoming increasingly employed, culture remains the gold standard for diagnosing periprosthetic joint infections. However, guidance for laboratory protocols is limited and highly variable. This article aims to increase diagnostic efficiency by providing concrete recommendations for medical microbiology laboratories.

## INTRODUCTION

Periprosthetic joint infection (PJI) is a serious complication of total joint replacement surgery, necessitating revision surgery and extended antibiotic therapy. Although the reported incidence of PJI in hip and knee replacements is currently at 1%–3% (1, 2), these rates are expected to rise due to population aging and the increasing number of total joint replacement surgeries performed (2–4). Timely microbiological diagnosis is crucial for effective treatment and optimal clinical outcomes. As PJI significantly affects the patient’s overall health and quality of life, it is essential to reduce the time to diagnosis (TTD).

The current gold standard for microbiological diagnosis of PJI is culture (5–7). Isolating the causative pathogen through culture can be challenging. This difficulty is mainly due to the diverse range of pathogens involved, including bacteria that are part of the normal skin flora, e.g., anaerobic bacteria like *Cutibacterium acnes* (1, 8). Consequently, these commensal bacteria can also be encountered as contaminants, further complicating PJI diagnosis. Moreover, bacteria residing within the biofilm on the prosthesis are in an inactive state, rendering cultivation more difficult (9).

In the past two decades, many efforts have been made to optimize the sensitivity and specificity of PJI culture. One strategy involves culturing multiple intraoperatively obtained samples, mainly aimed at distinguishing pathogens from contaminants (10). Another approach involves extending the incubation period for up to 2 weeks, which has led to improvements in culture yield (11, 12). Additionally, the use of enriched liquid media, such as broths and blood culture bottles (BCBs) (13–16), facilitates the growth of fastidious microorganisms and those present in low concentrations. Sonication fluid culture has also emerged as a widely adopted technique (1, 17–19) since it is presumed to represent the biofilm present on the affected prosthetic joint (20). Nevertheless, its utility remains a topic of debate, with no clear added benefit established over other sample types (19, 21–23).

Diverse culture protocols are employed across laboratories, but the prevailing consensus is that all PJI cultures should be incubated using enriched liquid media for a duration of 10–14 days (1, 17). Prolonged incubation periods may, however, introduce the risk of false-positive results (17, 24) and potential delays in the initialization of optimal antimicrobial treatment (25). Such delays can contribute to unfavorable clinical outcomes, prolonged hospital stays, and the development of antimicrobial resistance (AMR). To minimize TTD, it is therefore imperative to investigate which additional factors influence the duration of PJI culture.

A growing body of evidence suggests that a shorter incubation period of PJI culture is feasible, depending on the PJI category. For example, certain studies examining the time to positivity of pathogens have indicated that those involved in acute PJIs exhibit a shorter TTD compared to pathogens in chronic PJIs (26, 27). This difference may be attributed to the virulence of microorganisms associated with acute PJIs, such as *Staphylococcus aureus*, which often demonstrate a higher replication rate (1, 28). Chronic PJIs are more frequently associated with indolent microorganisms (14, 28). Consequently, some authors have concluded that the TTD of acute PJIs is shorter compared to that of chronic PJIs (26, 27), whereas others have found no such difference across PJI categories (29).

This study aims to determine the TTD of hip and knee PJIs, hypothesizing a significant difference in TTD between acute and chronic PJIs. Additionally, it aims to evaluate the culture yield of different enriched liquid media and assess the diagnostic utility of sonication fluid culture in confirming PJI diagnosis.

## MATERIALS AND METHODS

### Study population

This retrospective study included patients who had undergone partial or total revision surgery of hip or knee prosthetic joints at the Maastricht University Medical Center (MUMC+) between February 2019 and December 2023. MUMC+ is a PJI referral center in the region. A minimum of three samples taken during surgery was required. Culture-negative samples and samples from second-stage reimplantation surgery were excluded. Microbiological laboratory results and electronic patient records were analyzed. Retrospective research is exempt from the Dutch law on Medical Research (WMO). All data used for analysis were de-identified, coded, and analyzed anonymously. Consequently, formal informed consent from patients was not required. However, patients undergoing surgery for PJI were asked for permission to use their coded data for research purposes. Those who declined were excluded from the study.

### Laboratory procedures

Each sample set of an individual patient typically included one implant material, three tissue specimens, and one synovial fluid sample (Fig. S1). Samples were either transported to the microbiological laboratory during working hours (8 a.m. to 17:30 p.m. on weekdays, 8 a.m. to 17 p.m. on weekends) or refrigerated at 4°C. Implant materials were sonicated for 1 minute at 40 kHz in sterile Ringer lactate using an ultrasonic bath (Bactosonic BS 14; Bandelin GmbH, Berlin, Germany), and tissues were homogenized using a disperser (ULTRA-TURRAX Tube Drive; IKA GmbH, Staufen, Germany).

Homogenized tissue (10 µL), synovial fluid (10 µL), and sonication fluid (100 µL) were cultured on three solid culture media: blood agar (Becton Dickinson, Franklin Lakes, NJ, USA) and chocolate agar (Oxoid Ltd., Basingstoke, UK) under aerobic conditions with 5% CO_2_, and Schaedler agar (BD) under anaerobic conditions. Agars were incubated for 2 days at 35°C and examined on days 1 and 2. All samples were also cultured using two enriched liquid media: thioglycolate broth (bioMérieux, Marcy l'Etoile, France) and blood culture bottles (BD). Homogenized tissue samples and synovial fluid were cultured in thioglycolate broth and a pediatric blood culture bottle (1 mL each), while sonication fluid was cultured in thioglycolate broth (3–4 mL) and an anaerobic blood culture bottle (8–10 mL). All enriched liquid media were incubated for 14 days or until they exhibited signs of positivity. Thioglycolate broths were inspected daily on weekdays and were considered positive if they appeared visually cloudy. Then, they were subcultured on blood and Schaedler agar if the initial direct culture was negative. After 14 days, all thioglycolate broths underwent further subculturing on Schaedler agar and were incubated for an additional 2 days to ensure no anaerobic bacteria were missed. Blood culture bottles were subcultured on blood and Schaedler agar upon detection of positivity signaled by the automatic blood culture system (BACTEC FX; Becton Dickinson).

All morphologically distinct colonies underwent species identification using matrix-assisted laser desorption ionization-time of flight mass spectrometry (VITEK MS, bioMérieux). Antimicrobial susceptibility testing (AST) was conducted on each of these colonies. AST was performed using a VITEK2 instrument (bioMérieux), via disk diffusion (Rosco, Albertslund, Denmark) or ETEST (bioMérieux) according to the local laboratory protocol. The interpretation of AST was based on the clinical breakpoints defined by the European Committee on Antibiotic Susceptibility Testing (30).

### Definitions and analysis

Confirmed PJIs were determined based on the diagnostic criteria of the European Bone and Joint Infection Society (5). Definitive microbiological criteria were established as follows: either culture growth of >50 colony-forming units (CFUs) per milliliter of any microorganism from sonication fluid or culture growth of the same microorganism in two or more samples obtained from intraoperative synovial fluid and/or tissue samples. In our study, sonication fluid samples with growth <50 CFU/mL or growth exclusively from enriched liquid media were also included alongside intraoperative synovial fluid and/or tissue samples to assess whether microbiological criteria were met. In instances where the microbiological criteria were not met, electronic patient records were reviewed for additional confirmatory criteria, such as the presence of a sinus tract. If no additional confirmatory criteria were identified, the isolate was considered a contaminant, unless otherwise determined by the multidisciplinary team (MDT), consisting of clinical microbiologists and orthopedic surgeons. This approach mirrors real-world clinical practices. Confirmed PJIs were further categorized into three categories: early acute (EA), late acute (LA), and late chronic (LC) (Table 1) (31–33).

**TABLE 1:** PJI categories

Category	Definition	Symptom duration
Early acute (post-operative) PJI	A periprosthetic joint infection occurring within 3 months after the index arthroplasty	
Late acute (hematogenous) PJI	A periprosthetic joint infection occurring more than 3 months after the index arthroplasty; presenting with a sudden, acute onset of symptoms in a prior asymptomatic joint	Less than 4 weeks
Late chronic PJI	A periprosthetic joint infection occurring more than 3 months after the index arthroplasty; presenting with chronic pain and with or without loosening of the prosthesis	More than 4 weeks

TTD was calculated as the duration between the surgery date and the date of species determination, provided that the definitive microbiological criteria were met. For polymicrobial infections, TTD was equal to the TTD of the pathogen with the longest TTD. For each set of samples from an individual patient, three aspects were assessed: the type of sample that yielded positive-culture results, the number of positive samples per sample type, and the confirmation of diagnosis through culture growth from either solid or enriched liquid media. If the confirmation relied on growth from enriched liquid media, the specific media that showed growth (thioglycolate broth, blood culture bottle, or both) were recorded.

Statistical analysis was performed using IBM SPSS Statistics (version 28, Chicago, USA) software. Non-parametric tests were used to compare continuous and categorical variables among groups. The Mann-Whitney *U* test was used for comparisons between two groups, while the Kruskal-Wallis test was used for comparisons involving more than two groups. A *P*-value of 0.05 was considered statistically significant.

## RESULTS

### Study population

A total of 187 patients with confirmed PJI were included, comprising 115 hip PJIs and 72 knee PJIs. These PJI cases were categorized into 68 EA, 52 LA, and 67 LC PJIs. Monomicrobial infections predominated, accounting for 159 cases (85.0%). Polymicrobial infections were identified in 28 cases (15.0%), with 20 of these cases involving PJIs with two true pathogens. In total, 42 distinct bacterial species and 2 distinct *Candida* species were isolated, comprising 224 unique true pathogens (non-contaminant isolates) and 27 contaminants. Overall, *Staphylococcus aureus* and *Staphylococcus epidermidis* were the most frequently identified bacterial species (Table S1).

### Time to diagnosis

#### TTD differs between (late) acute and late chronic PJIs

**Fig 1:**
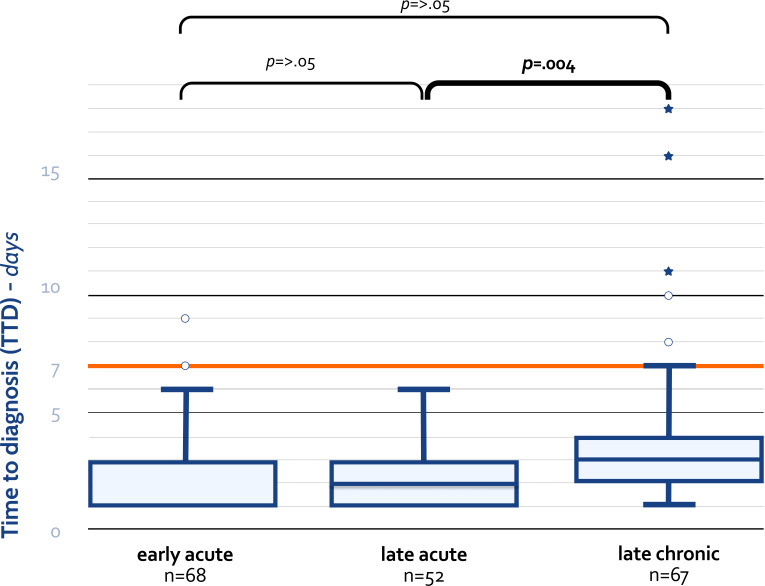
Time to diagnosis (days) per PJI category. Box plots depicting the distribution of time to diagnosis per PJI category. The interquartile range is equal to 2 across categories. For the early acute category, the median (3) coincides with the 75th percentile. ○, outlier (1.5–3 box lengths from 75th percentile); *, extreme outlier (>3 box lengths from 75th percentile).

Analysis of TTD using the Kruskal-Wallis test revealed a significant difference across the three PJI categories (χ^2^ [2, *n* = 187] = 12.07, *P* = 0.002), with a median TTD of 2 days for LA PJI and a median TTD of 3 days for EA and LC PJI. Subsequent Bonferroni *post hoc* comparisons indicated a significant difference in the median TTD between the LA and LC categories (*P* = 0.004) (Fig. 1). The majority of PJI cases (75.7%) were diagnosed within 5 days and 83.2% within 7 days (Fig. S2). When distinguishing between PJI categories, we observed that 98.5% of EA and 100% of LA PJI cases were diagnosed within 7 days, whereas this proportion decreased to 88.1% for LC PJI. TTD exceeded 7 days in nine cases (Table 2). Notably, eight out of these nine cases belonged to the LC category. Only one case had received antibiotics in the 2 weeks prior to surgery, which could have influenced culture results. Prolonged incubation periods of 10 and 14 days allowed diagnosis of 94% and 97% of LC PJI, respectively.

**TABLE 2:** PJI with TTD exceeding 7 days

Category PJI	Affected joint	TTD (days)	Pathogen	Number of positive samples per total number of samples	Confirmatory criteria
EA	Hip	9	*Staphylococcus epidermidis*	1/3	Decision of MDT
			*Micrococcus luteus*	1/3	Decision of MDT
LC	Knee	8	*Granulicatella adiacens*	2/3	≥2 samples with growth of same microorganism
	Hip	8	*Cutibacterium acnes*	1/7	Decision of MDT
	Hip	8	*Cutibacterium acnes*	2/3	≥2 samples with growth of same microorganism
	Hip	10	*Cutibacterium acnes*	3/5	≥2 samples with growth of same microorganism
			*Abiotrophia defectiva*	1/5	Decision of MDT
	Knee	11	*Cutibacterium acnes*	2/7	≥2 samples with growth of same microorganism
	Hip	11	*Cutibacterium avidum*	3/5	≥2 samples with growth of same microorganism
	Hip	16	*Cutibacterium avidum*	2/6	≥2 samples with growth of same microorganism
			*Bacillus cereus*	1/6	Decision of MDT
	Knee	18	*Staphylococcus warneri*	1/7	Decision of MDT

#### TTD differs between mono- and polymicrobial infections

Mann-Whitney *U* analysis demonstrated TTD was significantly shorter for monomicrobial PJI cases (*Md* = 2 and *n* = 159) compared to polymicrobial PJI cases (*Md* = 4, *n* = 28, U = 1,182.5, *z* = −4.06, and *P* ≤ 0.001). In total, 81.2% of all polymicrobial PJIs had a TTD of 5 days or less and 89.7% had a TTD of 7 days or less. The majority of polymicrobial PJI (71.4%) belonged to either the EA or LA category.

#### TTD differs between pathogens and contaminants

In a subgroup of PJI (*n* = 22, 11.7%), part of the detected isolates were identified as true pathogens, while some additional isolates were categorized as contaminants (Table S2). Both true pathogens and contaminants had a median TTD of 3 days; however, Mann-Whitney *U* analysis revealed that contaminants within true PJI cases had a significantly longer TTD compared to the true pathogens (*U* = 2,037, *z* = −2.84, *r* = −0.2, and *P* = 0.004).

### Enriched liquid media

#### Monomicrobial PJI

We observed 43 out of 159 (27.0%) monomicrobial PJIs, comprising a total of 237 individual culture samples, where either no pathogen growth was detected on solid media, or where only one sample showed growth on such media. Consequently, the confirmation of the microbiological PJI diagnosis relied on, or was aided by, the growth observed in broths and/or BCBs. Among these cases, a total of 46 individual culture samples showed growth solely from the anaerobic or pediatric BCB, while another 47 individual culture samples showed growth from both thioglycolate broth and BCB. No cases showed growth solely from thioglycolate broth.

#### Polymicrobial PJI

Upon using the same approach to analyze polymicrobial infections, we identified 4 out of 28 cases where all true pathogens were identified through direct culture results. In all other cases (*n* = 24, 85.7%), comprising a total of 141 individual culture samples and 65 individual pathogens, the majority of these pathogens (*n* = 45, 69.2%) were identified through the growth of enriched liquid media. Upon closer inspection, we identified six unique pathogens (comprising eight individual samples) that exclusively grew from thioglycolate broth, across five unique PJI. In the remaining cases (*n* = 19), growth was observed from either both enriched liquid media (29 individual samples) or solely from the BCB (39 individual samples).

#### Anaerobic PJI

We identified 11 PJI cases (5.9%) with growth of at least one anaerobic bacterium. Among these cases, *Cutibacterium acnes* was the most frequently identified bacterial species (*n* = 6). In four cases involving anaerobic bacteria, the confirmative diagnosis relied on growth from solid media. Conversely, in the seven remaining cases, growth occurred in at least one enriched medium: two cases exclusively from thioglycolate broth, two cases exclusively from BCB, and three cases from both enriched liquid media (Table S3).

### Sonication fluid culture

We identified 33 PJI cases (17.6%) where the diagnosis relied on growth from sonication culture, encompassing a total of 35 unique pathogens. *Staphylococcus epidermidis* was the most frequently identified pathogen (*n* = 10). Of these cases, 23 pathogens grew solely from sonication culture, meeting the threshold of >50 CFU/mL growth on solid agar plates. For the 12 remaining pathogens, the sonication culture served as the secondary sample to confirm the presence of the same microorganism, thereby meeting confirmatory microbiological criteria. All of these 12 pathogens were detected through either growth from BCB or growth from both enriched liquid media. Among the 33 cases, 11 were polymicrobial PJI.

## DISCUSSION

### Time to diagnosis of PJI

The primary aim of the present study was to determine the TTD of acute and chronic hip and knee PJI and explore the implications for culture optimization. Our findings corroborate the results of previous research indicating that the TTD of chronic PJIs surpasses that of acute PJIs (26, 27). With the exception of one case, all acute PJIs were diagnosed within 7 days. This is consistent with the data obtained by other scholars. Specifically, two studies showed that acute PJIs can be diagnosed in 5 days or less, whereas chronic PJIs can take longer (maximum 8 and 11 days, respectively) (26, 27). Due to their small sample size (*n* = 59 and *n* = 40, respectively), no definitive recommendations were made about the incubation period for acute and chronic PJI. In contrast, a large multicenter cohort (*n* = 183) did not find a significant difference in TTD between acute and chronic PJIs (29). Based on their findings, the authors concluded that empiric antibiotic therapy can be safely re-evaluated on day 5 for almost all PJI cases. Nevertheless, two out six cases with a TTD exceeding 5 days involved *Cutibacterium acnes* (TTD of 7 and 14 days, respectively). This bacterial species is known to require longer incubation periods and anaerobic growth conditions. Additionally, as a skin commensal, it can complicate the differentiation between true infection and contamination. In our cohort, we identified six LC cases with *Cutibacterium* spp. and a TTD exceeding 7 days (Table 2), with a maximal TTD of 16 days. Our findings emphasize the general agreement advocating for prolonged incubation periods to detect infections involving *Cutibacterium* spp. (8, 12).

In our analysis of TTD across PJI categories, we demonstrated a statistically significant difference between the LA and LC categories. It is worth noting that both PJI categories include infections occurring 3 months after index arthroplasty, yet they present with distinct clinical features and symptom duration. Building upon our findings and the similar outcomes reported by other researchers (26, 27), we propose a 7-day incubation period for acute PJI (either EA or LA) and to maintain a longer incubation period of 14 days for chronic PJI. In contrast to assertions made by other researchers (17) about the impracticality of implementing distinct incubation protocols for acute and chronic infections, we argue that this presents a unique opportunity to optimize PJI diagnosis. To implement this diagnostic strategy effectively, the orthopedic surgeon needs to provide accurate information regarding the clinical presentation to the medical microbiology laboratory in order to differentiate between LA and LC PJI. Electronic laboratory request forms, coupled with a diagnostic algorithm, could serve as an efficient gateway to clinical implementation. We firmly believe that this approach offers numerous advantages. First and foremost, by decreasing the TTD of acute PJI by up to 7 days, the time to definitive treatment can be reduced: decisions regarding the alteration or cessation of intravenously administered broad-spectrum antimicrobial therapy can be made earlier. This, in turn, facilitates earlier discharge from the hospital and helps prevent the emergence of AMR by discontinuing inappropriate treatment. Altogether, the reduction in TTD leads to a decrease in healthcare-associated costs (34–36).

Polymicrobial infections represent the minority of all PJIs, with a reported incidence varying between 20% and 35% (1, 37). Risk factors include early onset infections (1, 28), the presence of a sinus tract, previous joint revisions, and age over 65 years (38). According to our findings, polymicrobial PJIs have a statistically longer TTD compared to monomicrobial PJIs. This difference may be explained by variations in the virulence of the associated microorganisms and microbial competition for nutrients. However, the majority (82.1%) of all polymicrobial PJIs had a TTD of 5 days or less. Most of these polymicrobial PJIs belonged to the EA and LA category. We can, therefore, conclude that prolonged culture duration does not necessarily equate to increased detection of polymicrobial PJIs, particularly in acute cases, aligning with prior research (24, 29).

When interpreting culture results, it is important to distinguish the growth of contaminants within monomicrobial infections from true polymicrobial infections. To the best of our knowledge, this is the first study examining the presence of contaminants within true monomicrobial infections. We identified 22 PJI cases where contaminants grew alongside true pathogens, with contaminants exhibiting a longer TTD compared to true pathogens. Although this difference in TTD was statistically significant, the effect size (*r*) was small according to Cohen’s criteria (39). Nevertheless, this finding highlights yet another challenge for clinicians in diagnosing PJI. In cases where the clinical significance of the detected pathogens is uncertain, clinical microbiologists are encouraged to explore treatment options suitable for all identified pathogens. However, it is crucial to prioritize optimal therapy for the most likely and virulent pathogen to avoid overtreatment and its associated risks.

### Diagnostic value of enriched liquid media

Enriched liquid media have long been employed in PJI culture to enhance pathogen detection (13, 15, 16, 23, 40, 41). However, considerable variability in the selection of media exists across laboratories (17). While some laboratories opt for brain heart infusion, others prefer thioglycolate broth. Additionally, the use of blood culture bottles further contributes to this diversity. On the one hand, anaerobic BCBs are predominantly favored due to their inherent capability to detect anaerobic bacteria. On the other hand, pediatric BCBs require smaller sample volumes, rendering them particularly advantageous when sample material is limited, which often occurs with synovial fluid samples. Nevertheless, their use is restrained by their limited ability to detect strictly anaerobic bacteria. In our laboratory, a pragmatic approach is adopted, wherein both types of BCBs are used. When sample volume is limited (e.g., tissue homogenates and synovial fluid), pediatric BCBs are employed. Conversely, when a larger sample volume is available, anaerobic BCBs are used (e.g., sonication fluid samples).

This study aimed to assess the diagnostic value of enriched liquid media, examining the necessity of their use in addition to agar plates and the requirement for combining broths and BCB. Our results reveal that the majority of monomicrobial PJIs were detected through growth on solid culture media, but 27% demanded the use of enriched liquid media to confirm the infection. Notably, thioglycolate broth did not offer diagnostic benefits in monomicrobial PJIs, as cases, where growth was detected from thioglycolate broth, showed growth from pediatric and/or anaerobic BCB as well. In contrast, in polymicrobial PJIs, we identified five cases where the causative pathogens were solely identified through growth from thioglycolate broth. Upon closer inspection of these cases, only one case met microbiological criteria based on growth from thioglycolate broth alone. This particular case was an LC PJI with *Cutibacterium acnes*. Thioglycolate broth is used for the detection of anaerobic bacteria and is subsequently an important tool for the detection of *Cutibacterium* spp. (41–46). Nonetheless, anaerobic PJIs in this cohort, including *Cutibacterium* spp*.,* were mostly isolated from anaerobic BCB, and even from pediatric BCB in some instances (Table S3).

In summary, enriched liquid media demonstrate a clear incremental benefit for diagnosing PJI alongside solid agar plates, particularly in cases of polymicrobial PJI and PJI involving anaerobic bacteria such as *Cutibacterium* spp. Thioglycolate broths appear to offer minimal cost-effectiveness in identifying causative pathogens. We, therefore, recommend medical microbiology laboratories to consider discontinuing their use when using an automatic blood culture system. Although BCBs are more costly than broths, we argue that their benefits justify the higher expense. Our findings indicate that BCBs provide higher sensitivity, supporting a reduction in time to diagnosis. Additionally, discontinuing thioglycolate broths reduces hands-on time for laboratory technicians as it eliminates the need for inoculation and daily inspection. When employing sonication fluid culture, larger sample volumes can be inoculated in anaerobic BCB. In scenarios where sample material is limited, pediatric BCBs offer a practical alternative.

### Diagnostic value of sonication fluid culture

Despite ongoing debate surrounding its utility, sonication has become a widely adopted technique in the diagnosis of biofilm-associated infections (1, 17). While some authors argue against its superiority in detecting PJI compared to other sample types (22, 23, 47), others assert its efficacy in enhancing detection (21, 48, 49). In our laboratory, sonication is routinely applied to prosthetic joints, and sonication fluid is subsequently cultured. We fervently advocate for the use of sonication fluid culture whenever feasible, as our findings demonstrate its indispensable role in diagnosis confirmation in a significant proportion (17.6%) of PJI cases. Interestingly, one-third of these cases were polymicrobial, highlighting its potential incremental benefits for the diagnosis of these infections. Furthermore, the majority of the causative pathogens were detected through growth from BCB, thereby emphasizing the utility of inoculation of sonication fluid in anaerobic BCB.

In conclusion, our results confirm that acute PJIs have a shorter TTD compared to chronic PJIs. Based on our findings, we propose a maximal incubation period of 7 days for acute PJI and 14 days for chronic PJI. Moreover, we recommend the implementation of sonication fluid culture to optimize culture sensitivity, particularly when combined with inoculation in anaerobic blood culture bottles as an enrichment medium. Thioglycolate broth confers marginal additional diagnostic benefit when using this approach. Taken together, this culture strategy can lead to a significant decrease in healthcare-associated costs. It is important to note that these findings are based solely on hip and knee PJIs and may not be applicable to shoulder PJIs, where *Cutibacterium acnes* is a common pathogen and requires extended incubation periods. To ensure no pathogens are missed, further research should focus on the implementation of culture-independent techniques (e.g., molecular diagnostics) for cases where conventional culture results remain negative on day 7 and day 10 for acute and chronic PJIs, respectively.

## SUPPLEMENTAL MATERIAL

Supplemental material jcm.01143-24-s0001.docx Tables S1 to S3; Figures S1 and S2.

ASM does not own the copyrights to Supplemental Material that may be linked to, or accessed through, an article. The authors have granted ASM a non-exclusive, world-wide license to publish the Supplemental Material files. Please contact the corresponding author directly for reuse.
