# Supplementary material for: Prolonged incubation time unwarranted for acute periprosthetic joint infections
Source: J Clin Microbiol. 2025 Jan 16;63(2):e01143-24. doi: 10.1128/jcm.01143-24 (PMC11837491; doi:10.1128/jcm.01143-24)
Supplement: Supplemental material — Tables S1 to S3; Figures S1 and S2. [file jcm.01143-24-s0001.docx]

**SUPPLEMENTAL MATERIAL**

**Table S1.A Identified microbial species - All PJI (*n*=187)**

|  | **Species** | **Total**  Count (%) | **True pathogen**  Count (%) | **Contaminant**  Count (%) |
| --- | --- | --- | --- | --- |
|  | *Staphylococcus aureus* | 61 (32,6) | 61 (32,6) | 0 (0,0) |
|  | *Staphylococcus epidermidis* | 53 (28,3) | 47 (25,1) | 6 (3,2) |
|  | *Streptococcus dysgalactiae* | 14 (7,5) | 13 (7,0) | 1 (0,5) |
|  | *Staphylococcus lugdunensis* | 12 (6,4) | 12 (6,4) | 0 (0,0) |
|  | *Enterococcus faecalis* | 10 (5,3) | 10 (5,3) | 0 (0,0) |
|  | *Streptococcus agalactiae* | 8 (4,3) | 8 (4,3) | 0 (0,0) |
|  | *Escherichia coli* | 7 (3,7) | 7 (3,7) | 0 (0,0) |
|  | *Cutibacterium acnes* | 7 (3,7) | 6 (3,2) | 1 (0,5) |
|  | *Staphylococcus haemolyticus* | 6 (3,2) | 5 (2,7) | 1 (0,5) |
|  | *Proteus mirabilis* | 5 (2,7) | 4 (2,1) | 1 (0,5) |
|  | *Staphylococcus warneri* | 5 (2,7) | 3 (1,6) | 2 (1,1) |
|  | *Streptococcus mitis group* | 5 (2,7) | 5 (2,7) | 0 (0,0) |
|  | *Streptococcus sanguinis group* | 5 (2,7) | 2 (1,1) | 3 (1,6) |
|  | *Enterococcus faecium* | 4 (2,1) | 4 (2,1) | 0 (0,0) |
|  | *Enterobacter cloacae complex* | 4 (2,1) | 4 (2,1) | 0 (0,0) |
|  | *Staphylococcus capitis* | 4 (2,1) | 3 (1,6) | 1 (0,5) |
|  | *Cutibacterium avidum* | 3 (1,6) | 3 (1,6) | 0 (0,0) |
|  | *Granulicatella adiacens* | 3 (1,6) | 3 (1,6) | 0 (0,0) |
|  | *Streptococcus anginosus group* | 3 (1,6) | 3 (1,6) | 0 (0,0) |
|  | *Pseudomonas aeruginosa* | 3 (1,6) | 3 (1,6) | 0 (0,0) |
|  | *Klebsiella pneumoniae* | 2 (1,1) | 2 (1,1) | 0 (0,0) |
|  | *Serratia marcescens* | 2 (1,1) | 2 (1,1) | 0 (0,0) |
|  | *Ralstonia insidiosa* | 2 (1,1) | 0 (0,0) | 2 (1,1) |
|  | *Staphylococcus hominis* | 2 (1,1) | 1 (0,5) | 1 (0,5) |
|  | *Streptococcus pyogenes* | 2 (1,1) | 2 (1,1) | 0 (0,0) |
|  | *Bacillus cereus* | 1 (0,5) | 1 (0,5) | 0 (0,0) |
|  | *Bacillus fordii* | 1 (0,5) | 0 (0,0) | 1 (0,5) |
|  | *Abiotrophia defectiva* | 1 (0,5) | 1 (0,5) | 0 (0,0) |
|  | *Candida albicans* | 1 (0,5) | 1 (0,5) | 0 (0,0) |
|  | *Candida parapsilosis* | 1 (0,5) | 0 (0,0) | 1 (0,5) |
|  | *Roseomonas mucosa* | 1 (0,5) | 0 (0,0) | 1 (0,5) |
|  | *Citrobacter koseri* | 1 (0,5) | 1 (0,5) | 0 (0,0) |
|  | *Rothia species* | 1 (0,5) | 0 (0,0) | 1 (0,5) |
|  | *Streptococcus salivarius group* | 1 (0,5) | 0 (0,0) | 1 (0,5) |
|  | *Acinetobacter pittii* | 1 (0,5) | 0 (0,0) | 1 (0,5) |
|  | *Gemella haemolysans* | 1 (0,5) | 1 (0,5) | 0 (0,0) |
|  | *Finegoldia magna* | 1 (0,5) | 1 (0,5) | 0 (0,0) |
|  | *Enterobacter aerogenes* | 1 (0,5) | 1 (0,5) | 0 (0,0) |
|  | *Clostridium perfringens* | 1 (0,5) | 1 (0,5) | 0 (0,0) |
|  | *Staphylococcus simulans* | 1 (0,5) | 1 (0,5) | 0 (0,0) |
|  | *Staphylococcus sciuri* | 1 (0,5) | 1 (0,5) | 0 (0,0) |
|  | *Corynebacterium simulans* | 1 (0,5) | 0 (0,0) | 1 (0,5) |
|  | *Staphylococcus lentus* | 1 (0,5) | 0 (0,0) | 1 (0,5) |

**Table S1.B True pathogens per PJI category**

|  | **Species** | **All PJI (*n*=187)**  Count (%) | **EA (*n*=68)**  Count (%) | **LA (*n*=52)**  Count (%) | **LC (*n*=67*)***  Count (%) |
| --- | --- | --- | --- | --- | --- |
|  | *Staphylococcus aureus* | 61 (32,6) | 30 (44,1) | 24 (46,2) | 7 (10,4) |
|  | *Staphylococcus epidermidis* | 47 (25,1) | 18 (26,5) | 8 (15,4) | 21 (31,3) |
|  | *Streptococcus dysgalactiae* | 13 (7,0) | 5 (7,4) | 6 (11,5) | 2 (3,0) |
|  | *Staphylococcus lugdunensis* | 12 (6,4) | 3 (4,4) | 3 (5,7) | 6 (9,0) |
|  | *Enterococcus faecalis* | 10 (5,3) | 6 (8,8) | 2 (3,8) | 2 (3,0) |
|  | *Streptococcus agalactiae* | 8 (4,3) | 3 (4,4) | 4 (7,7) | 1 (1,5) |
|  | *Escherichia coli* | 7 (3,7) | 2 (3,0) | 2 (3,8) | 3 (4,5) |
|  | *Cutibacterium acnes* | 6 (3,2) | 0 (0,0) | 0 (0,0) | 6 (9,0) |
|  | *Streptococcus mitis group* | 5 (2,7) | 2 (3,0) | 2 (3,8) | 1 (1,5) |
|  | *Staphylococcus haemolyticus* | 5 (2,7) | 4 (5,9) | 0 (0,0) | 1 (1,5) |
|  | *Proteus mirabilis* | 4 (2,1) | 2 (3,0) | 0 (0,0) | 2 (3,0) |
|  | *Enterococcus faecium* | 4 (2,1) | 2 (3,0) | 1 (1,9) | 1 (1,5) |
|  | *Enterobacter cloacae complex* | 4 (2,1) | 2 (3,0) | 1 (1,9) | 1 (1,5) |
|  | *Staphylococcus warneri* | 3 (1,6) | 1 (1,5) | 0 (0,0) | 2 (3,0) |
|  | *Staphylococcus capitis* | 3 (1,6) | 0 (0,0) | 1 (1,9) | 2 (3,0) |
|  | *Cutibacterium avidum* | 3 (1,6) | 0 (0,0) | 0 (0,0) | 3 (4,5) |
|  | *Granulicatella adiacens* | 3 (1,6) | 0 (0,0) | 0 (0,0) | 3 (4,5) |
|  | *Streptococcus anginosus group* | 3 (1,6) | 0 (0,0) | 0 (0,0) | 3 (4,5) |
|  | *Pseudomonas aeruginosa* | 3 (1,6) | 2 (3,0) | 0 (0,0) | 1 (1,5) |
|  | *Streptococcus sanguinis group* | 2 (1,1) | 0 (0,0) | 1 (1,9) | 1 (1,5) |
|  | *Klebsiella pneumoniae* | 2 (1,1) | 1 (1,5) | 0 (0,0) | 1 (1,5) |
|  | *Serratia marcescens* | 2 (1,1) | 1 (1,5) | 0 (0,0) | 1 (1,5) |
|  | *Streptococcus pyogenes* | 2 (1,1) | 2 (3,0) | 0 (0,0) | 0 (0,0) |
|  | *Staphylococcus hominis* | 1 (0,5) | 1 (1,5) | 0 (0,0) | 0 (0,0) |
|  | *Bacillus cereus* | 1 (0,5) | 0 (0,0) | 0 (0,0) | 1 (1,5) |
|  | *Abiotrophia defectiva* | 1 (0,5) | 0 (0,0) | 0 (0,0) | 1 (1,5) |
|  | *Candida albicans* | 1 (0,5) | 1 (1,5) | 0 (0,0) | 0 (0,0) |
|  | *Citrobacter koseri* | 1 (0,5) | 0 (0,0) | 1 (1,9) | 0 (0,0) |
|  | *Gemella haemolysans* | 1 (0,5) | 0 (0,0) | 0 (0,0) | 1 (1,5) |
|  | *Finegoldia magna* | 1 (0,5) | 0 (0,0) | 1 (1,9) | 0 (0,0) |
|  | *Enterobacter aerogenes* | 1 (0,5) | 0 (0,0) | 0 (0,0) | 1 (1,5) |
|  | *Clostridium perfringens* | 1 (0,5) | 1 (1,5) | 0 (0,0) | 0 (0,0) |
|  | *Staphylococcus simulans* | 1 (0,5) | 1 (1,5) | 0 (0,0) | 0 (0,0) |
|  | *Staphylococcus sciuri* | 1 (0,5) | 0 (0,0) | 1 (1,9) | 0 (0,0) |

*EA= early acute, LA= late acute, LC= late chronic*

**Table S2. PJI with contaminants within true infection**

| **Class** | **Affected joint** | **True pathogen** | **TTD** | **Number of positive samples per total number of samples** | **Contaminant** | **TTD** | **Number of positive samples per total number of samples** | **Sample type of positive sample(s)** | **Culture positive media** |
| --- | --- | --- | --- | --- | --- | --- | --- | --- | --- |
| EA | hip | *Enterococcus faecalis* | 1 | 3/7 | *Staphylococcus epidermidis* | 1 | 1/7 | 1/5 tissues | BCB and THIO |
|  | hip | *Staphylococcus epidermidis* | 5 | 3/6 | *Proteus mirabilis* | 2 | 1/6 | 1/2 SON | BCB |
|  | knee | *Staphylococcus aureus* | 1 | 10/11 | *Staphylococcus warneri* | 8 | 1/11 | 1/4 SON | BCB |
|  | knee | *Staphylococcus aureus* | 3 | 3/5 | *Acinetobacter pitii* | 3 | 1/5 | 1/2 tissues | BCB |
|  | hip | *Candida albicans* | 3 | 4/5 | *Staphylococcus epidermidis* | 3 | 1/5 | 1/3 tissues | BCB and THIO |
|  | knee | *Staphylococcus aureus* | 1 | 3/6 | *Streptococcus dysgalactiae* | 2 | 1/6 | 1/1 IMP | <50 cfu/mL on blood agar plate |
|  |  |  |  |  | *Corynebacterium simulans* | 5 | 1/6 | 1/4 tissues | BCB and THIO |
|  | hip | *Staphylococcus epidermidis* | 2 | 4/4 | *Staphylococcus hominis* | 2 | 1/4 | 1/3 tissues | BCB |
|  |  | *Staphylococcus aureus* | 4 | 1/4^*^ |  |  |  |  |  |
|  | hip | *Staphylococcus lugdunensis* | 3 | 3/4 | *Staphylococcus epidermidis* | 2 | 1/4 | 1/1 SON | BCB and THIO |
|  |  |  |  |  | *Staphylococcus lentus* | 2 | 1/4 | 1/1 SON | BCB and THIO |
| LA | knee | *Staphylococcus aureus* | 1 | 6/7 | *Staphylococcus epidermidis* | 5 | 1/7 | 1/3 tissues | BCB |
|  | hip | *Staphylococcus epidermidis* | 2 | 2/5 | *Roseomonas mucosa* | 3 | 1/5 | 1/4 tissues | BCB |
|  |  | *Staphylococcus aureus* | 3 | 1/5^*^ |  |  |  |  |  |
| LC | hip | *Escherichia coli* | 4 | 6/6 | *Streptococcus sanguinis group* | 4 | 1/6 | 1/2 SON | BCB |
|  | knee | *Granulicatella adiacens* | 8 | 2/3 | *Ralstonia insidiosa* | 3 | 1/3 | 1/1 SON | THIO |
|  | hip | *Granulicatella adiacens* | 4 | 1/4^¤^ | *Cutibacterium acnes* | 11 | 1/4 | 1/2 SON | BCB |
|  | knee | *Cutibacterium acnes* | 11 | 2/6 | *Bacillus fordii* | 11 | 1/6 | 1/4 tissues | BCB |
|  | hip | *Proteus mirabilis* | 3 | 3/6 | *Staphylococcus epidermidis* | 15 | 1/6 | 1/1 SON | THIO |
|  | hip | *Staphylococcus epidermidis* | 3 | 1/4^¤^ | *Ralstonia insidiosa* | 3 | 1/4 | 1/1 SON | < 50 cfu/mL on blood agar plate |
|  | knee | *Staphylococcus epidermidis* | 2 | 1/7* | *Candida parapsilosis* | 16 | 1/7 | 1/1 SYN | THIO |
|  | knee | *Staphylococcus lugdunensis* | 3 | 5/7 | *Staphylococcus haemolyticus* | 3 | 1/7 | 1/4 tissues | BCB |
|  | knee | *Staphylococcus epidermidis* | 3 | 1/8^*^ | *Staphylococcus warneri* | 3 | 1/8 | 1/1 SON | BCB |
|  | hip | *Granulicatella adiacens* | 6 | 4/4 | *Staphylococcus capitis* | 4 | 1/4 | 1/1 SON | BCB |
|  |  |  |  |  | *Staphylococcus epidermidis* | 4 | 1/4 | 1/3 tissues | BCB |
|  | hip | *Pseudomonas aeruginosa* | 2 | 2/5 | *Streptococcus salivarius group* | 2 | 1/5 | 1/1 SON | BCB |
|  |  |  |  |  | *Streptococcus sanguinis group* | 3 | 1/5 | 1/1 SON | BCB |
|  | hip | *Staphylococcus epidermidis* | 4 | 5/7 | *Rothia species* | 6 | 1/7 | 1/5 tissues | BCB |
|  |  | *Enterobacter cloacae complex* | 4 | 1/7^*^ | *Streptococcus sanguinis group* | 4 | 1/7 | 1/5 tissues | BCB |

*EA= early acute, LA= late acute, LC= late chronic, SON = sonication fluid sample, SYN = synovial fluid sample, THIO= thioglycolate broth, BCB= blood culture bottle*

*Confirmatory criteria of true pathogens: ^*^ Decision to treat as true pathogen made by multidisciplinary team.* ^¤^ *Growth of >50 cfu/mL on agar plate(s) in sonication fluid sample. All other cases: ≥2 samples with growth of same microorganism.*

**Table S3. Diagnosis of anaerobic PJI**

| **Category PJI** | **Pathogen** | **TTD (days)** | **Number of positive samples per total number of samples** | **Enriched liquid media** | **Confirmatory criteria** |
| --- | --- | --- | --- | --- | --- |
| EA | *Clostridium perfringens* | 3 | 3/5 |  | ≥2 samples with growth of same microorganism |
| LA | *Finegoldia magna* | 6 | 3/5 |  | ≥2 samples with growth of same microorganism |
| LC | *Cutibacterium acnes* | 4 | 1/7 |  | >50 CFU/ml of any organism in sonication culture |
|  |  | 7 | 1/7 | 1x THIO | Decision of MDT |
|  |  | 8 | 1/7 | 1x anaerobic BCB | Decision of MDT |
|  |  | 8 | 2/3 | 2x anaerobic BCB and THIO | ≥2 samples with growth of same microorganism |
|  |  | 10 | 3/5 | 3x THIO | ≥2 samples with growth of same microorganism |
|  |  | 11 | 2/7 | 1x anaerobic BCB and THIO  1x pediatric BCB | ≥2 samples with growth of same microorganism |
|  | *Cutibacterium avidum* | 3 | 6/7 |  | >50 CFU/ml of any organism in sonication culture |
|  |  | 11 | 3/5 | 1x anaerobic BCB  1x pediatric BCB | ≥2 samples with growth of same microorganism |
|  |  | 16 | 2/6 | 1x anaerobic BCB and THIO  1x pediatric BCB | ≥2 samples with growth of same microorganism |

*TTD=time to diagnosis, EA= early acute, LA= late acute, LC= late chronic, MDT=multidisciplinary team, THIO= thioglycolate broth, BCB= blood culture bottle*

**Figure S1. Distribution of number of intraoperatively collected samples per sample type.**

**
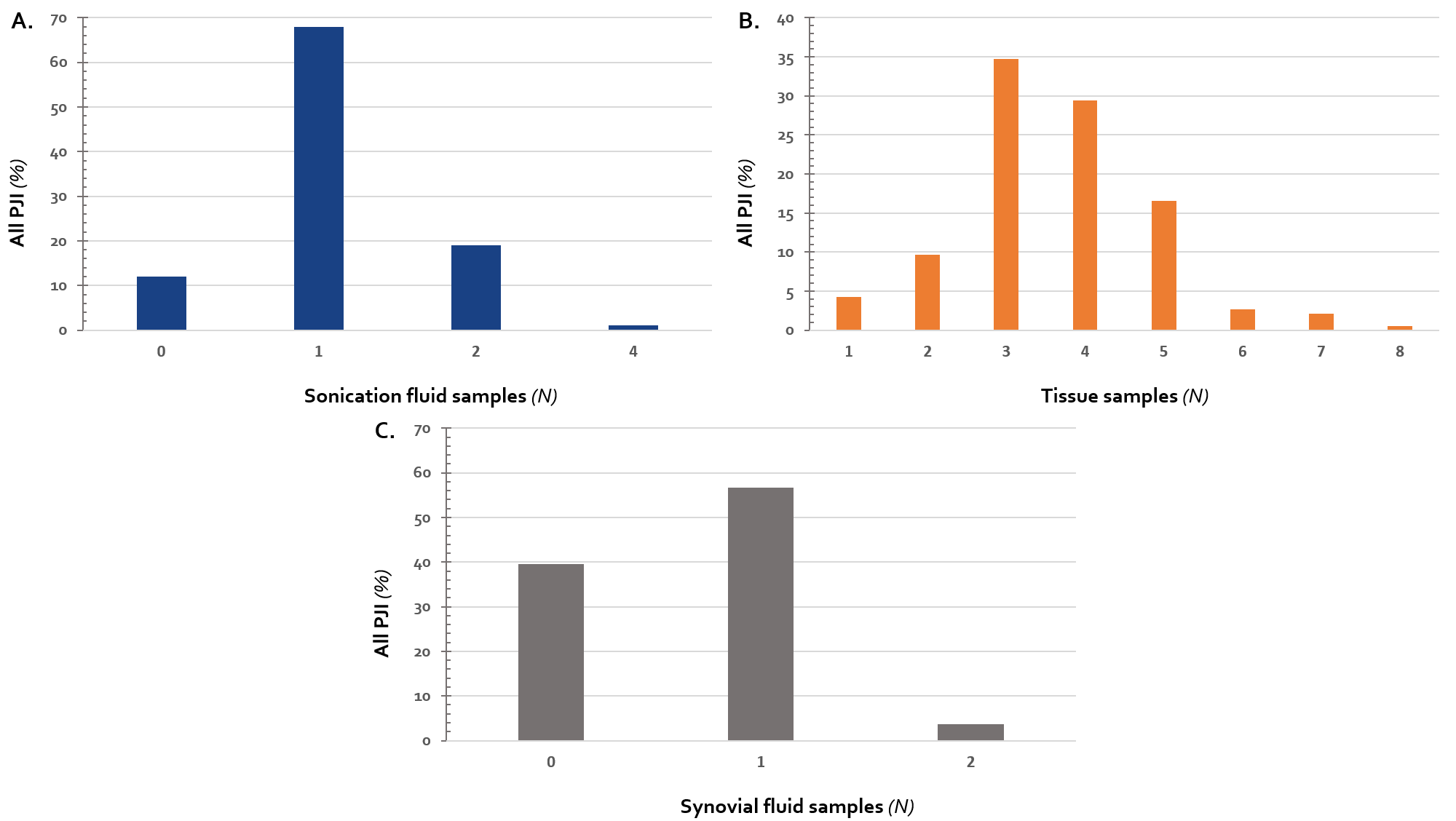
**

**Figure S2. Culture positivity rate per sample type and per day**

**
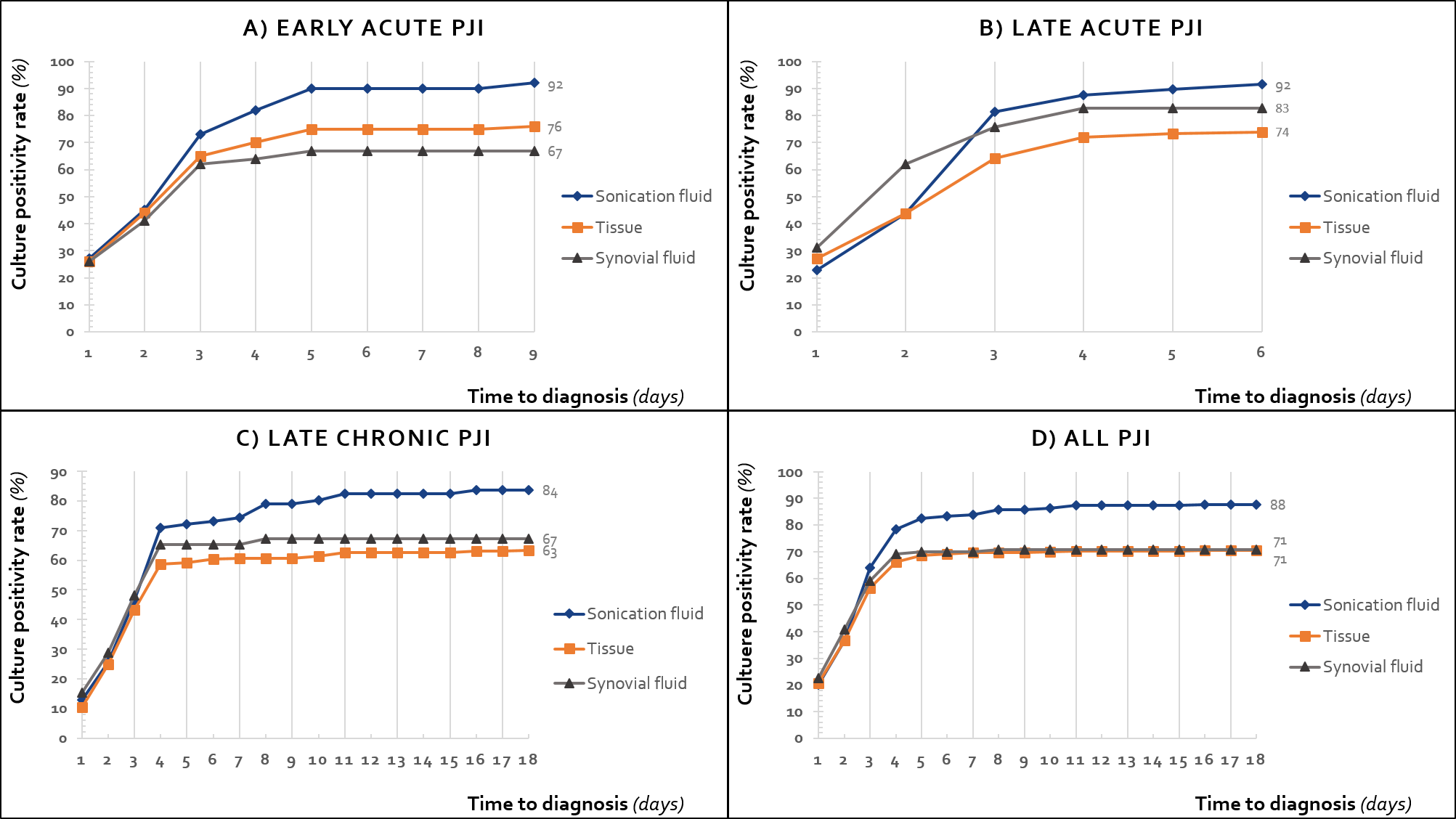
**
